# FABP5 deletion in nociceptors augments endocannabinoid signaling and suppresses TRPV1 sensitization and inflammatory pain

**DOI:** 10.1038/s41598-022-13284-0

**Published:** 2022-06-02

**Authors:** Diane M. Bogdan, Keith Studholme, Adriana DiBua, Chris Gordon, Martha P. Kanjiya, Mei Yu, Michelino Puopolo, Martin Kaczocha

**Affiliations:** 1 Department of Anesthesiology, Renaissance School of Medicine, Stony Brook University, Stony Brook, NY 11794 USA; 2 Stony Brook University Pain and Analgesia Research Center (SPARC), Renaissance School of Medicine, Stony Brook University, Stony Brook, NY 11794 USA

**Keywords:** Peripheral nervous system, Lipids, Neurochemistry, Preclinical research

## Abstract

The endocannabinoid anandamide (AEA) produces antinociceptive effects by activating cannabinoid receptor 1 (CB1). However, AEA also serves as an agonist at transient receptor potential vanilloid receptor 1 (TRPV1) in nociceptive sensory neurons, which may exacerbate pain. This potential functional duality is highlighted by the failure of an inhibitor of the AEA catabolic enzyme fatty acid amide hydrolase (FAAH) to afford pain relief in a clinical trial. Consequently, it remains to be determined whether elevating AEA levels in nociceptors leads to antinociceptive or pro-nociceptive effects. Fatty acid binding protein 5 (FABP5) is an intracellular carrier that mediates AEA transport to FAAH for inactivation. Leveraging the abundant expression of FABP5 in TRPV1^+^ nociceptors, we employed a conditional knockout strategy to demonstrate that FABP5 deletion in nociceptors augments AEA levels, resulting in the emergence of antinociceptive effects mediated by CB1. Mechanistically, FABP5 deletion suppresses inflammation- and nerve growth factor-mediated TRPV1 sensitization via CB1, an effect mediated by calcineurin. Unexpectedly, inhibition of FAAH failed to blunt TRPV1 sensitization, uncovering functionally distinct outputs resulting from FABP5 and FAAH inhibition. Collectively, our results demonstrate that FABP5 serves a key role in governing endocannabinoid signaling in nociceptors to disrupt TRPV1 sensitization and pain, and position FABP5 as a therapeutic target for the development of analgesics.

## Introduction

The endocannabinoid system has emerged as an attractive target for the development of analgesics. The endocannabinoids anandamide (AEA) and 2-arachidonoylglycerol (2-AG) are endogenous ligands for cannabinoid receptors 1 and 2 (CB1 and CB2, respectively), whose activation produces antinociceptive effects in diverse models of pain^1–3^. In the periphery, the CB1 receptor is expressed in nociceptive sensory neurons wherein it mediates the antinociceptive effects of phytocannabinoids^4–6^. Sensory neurons also express enzymes that catalyze AEA biosynthesis and inactivation^7,8^, indicative of a functional endocannabinoid system. Nociceptors express a host of ion channels that modulate pain including transient receptor potential vanilloid receptor 1 (TRPV1), a non-selective cation channel that is a central mediator of inflammatory pain^9,10^. During inflammation, TRPV1 undergoes sensitization that sustains hyperalgesia^11–14^ while disruption of TRPV1 sensitization promotes analgesia^15^. Therefore, proteins that modulate TRPV1 sensitization constitute promising targets for the development of analgesics^15–17^.

It is well-established that enhancement of peripheral AEA signaling via inhibition of its catabolic enzyme fatty acid amide hydrolase (FAAH) produces antinociceptive effects mediated by CB1 and/or CB2 receptors^18,19^. However, a clinical trial of a FAAH inhibitor failed to demonstrate clinically meaningful analgesia^20^. A plausible mechanism to explain this discrepancy stems from previous observations that FAAH substrates including AEA engage additional molecular targets that may counteract the antinociceptive effects resulting from CB1 activation. In addition to serving as a CB1 ligand, AEA is also a TRPV1 agonist and inflammatory mediators such as prostaglandin E2 increase its efficacy and potency at TRPV1, which may enhance hyperalgesia^21–24^. The structurally related FAAH substrate oleoylethanolamide (OEA) likewise serves as a potent TRPV1 agonist and can elicit pro-nociceptive effects in some settings^25^. Moreover, FAAH regulates the metabolism of N-acyl taurines that activate TRPV1^26^. Taken together, although FAAH inhibition produces antinociceptive effects in diverse preclinical models of pain, its inhibition simultaneously augments the levels of distinct classes of TRPV1 agonists. Consistent with this notion, FAAH knockout (KO) mice exhibit a pro-nociceptive phenotype characterized by exacerbated TRPV1 activation^27^.

Activation of TRPV1 in nociceptors stimulates the release of neurotransmitters and neuropeptides into the dorsal horn of the spinal cord, including the pro-nociceptive neuropeptide calcitonin gene-related peptide (CGRP) whose release is enhanced in the setting of chronic pain and is tempered by analgesics^28–30^. AEA exhibits biphasic activity wherein it inhibits TRPV1-mediated CGRP release from dorsal root ganglia (DRG) at low concentrations via CB1 while enhancing CGRP release and nociceptor output at higher concentrations^18,24,31–38^. Moreover, stimulation of AEA synthesis in DRG neurons promotes TRPV1 activation^8^, suggesting that interventions that increase AEA levels during inflammation could in principle exacerbate pain. Consequently, a major question that remains to be answered is whether augmenting AEA levels in TRPV1^+^ nociceptors leads to CB1-mediated antinociceptive effects or pro-nociceptive effects elicited by TRPV1 activation.

Fatty acid binding proteins (FABPs) are a family of intracellular carriers for endocannabinoids^39,40^. We previously identified FABP5 as the major FABP subtype that mediates AEA transport to FAAH for inactivation^39^. In addition to AEA, FABP5 also governs the transport of the related N-acylethanolamines OEA and palmitoylethanolamide (PEA), which serve as agonists at peroxisome proliferator-activated receptor alpha (PPARα)^41,42^. Consequently, pharmacological or genetic inhibition of FABP5 elevates AEA, PEA, and OEA levels and produces antinociceptive effects via CB1 and PPARα^43–45^. FABP5 is the predominant FABP subtype in sensory neurons and is extensively distributed in TRPV1^+^ nociceptors^45,46^. Similarly, CB1, PPARα, and FAAH are widely expressed in sensory neurons while robust AEA synthesis and ensuing TRPV1 activation are observed in TRPV1^+^ nociceptors *in vitro*^4,8,46,47^. The overlapping distribution between FABP5 and AEA metabolizing enzymes in TRPV1^+^ nociceptors provides a unique opportunity to leverage FABP5 deletion to augment AEA levels in nociceptors and in turn resolve outstanding questions underlying the functional role of AEA as a modulator of TRPV1 activation and inflammatory pain.

## Results

### Inflammatory hyperalgesia in FABP5 cKO mice

**Figure 1:**
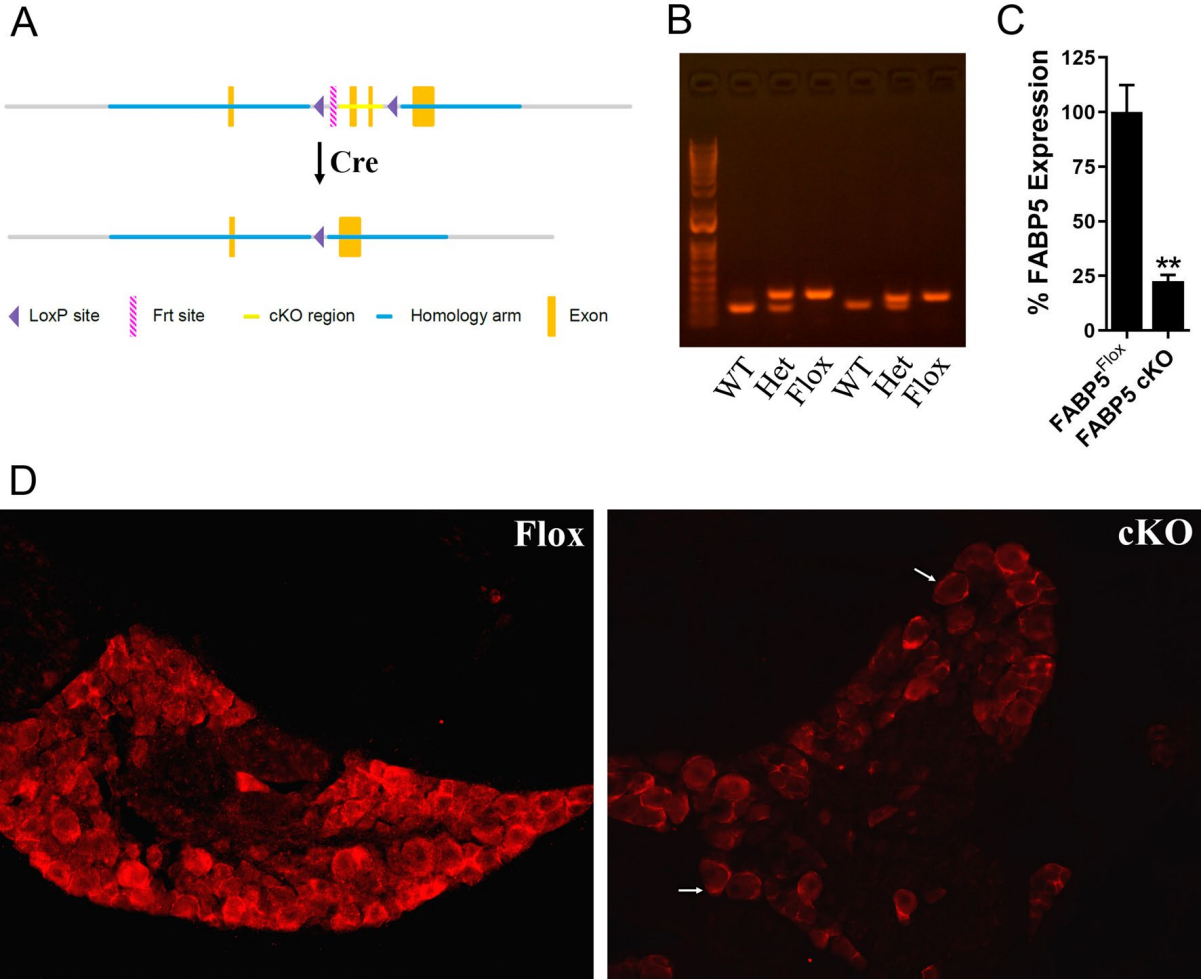
Characterization of FABP5 cKO mice. (**A**) Schematic showing the generation of FABP5^Flox^ mice and placement of LoxP sites. (**B**) PCR analysis of WT, heterozygous, and homozygous FABP5^Flox^ mice. (**C**) qPCR analysis of relative FABP5 expression in DRGs of FABP5^Flox^ and FABP5 cKO mice. **p < 0.01 calculated using unpaired *t* test (n = 3). (**D**) FABP5 distribution in DRGs of FABP5^Flox^ and FABP5 cKO mice.

FABP5^Flox^ mice were designed with loxP sites flanking exons 2 and 3 of the FABP5 gene (Fig. 1A,B). The mice were born at expected Mendelian frequencies and were otherwise normal. To ablate FABP5 in TRPV1^+^ nociceptors, we crossed FABP5^Flox^ mice with TRPV1-Cre mice to generate FABP5^Flox/TRPV1-Cre^ mice (hereafter referred to as FABP5 cKO). Reduced FABP5 expression in DRGs of FABP5 cKO mice was confirmed via qPCR and histologically (Fig. 1C,D, Supplementary Fig. 1). Note that residual FABP5 immunostaining in FABP5 cKO DRGs was observed in cells surrounding sensory neurons (Fig. 1D, arrowheads), presumably reflecting FABP5 expression in satellite glial cells^46^.

**Figure 2:**
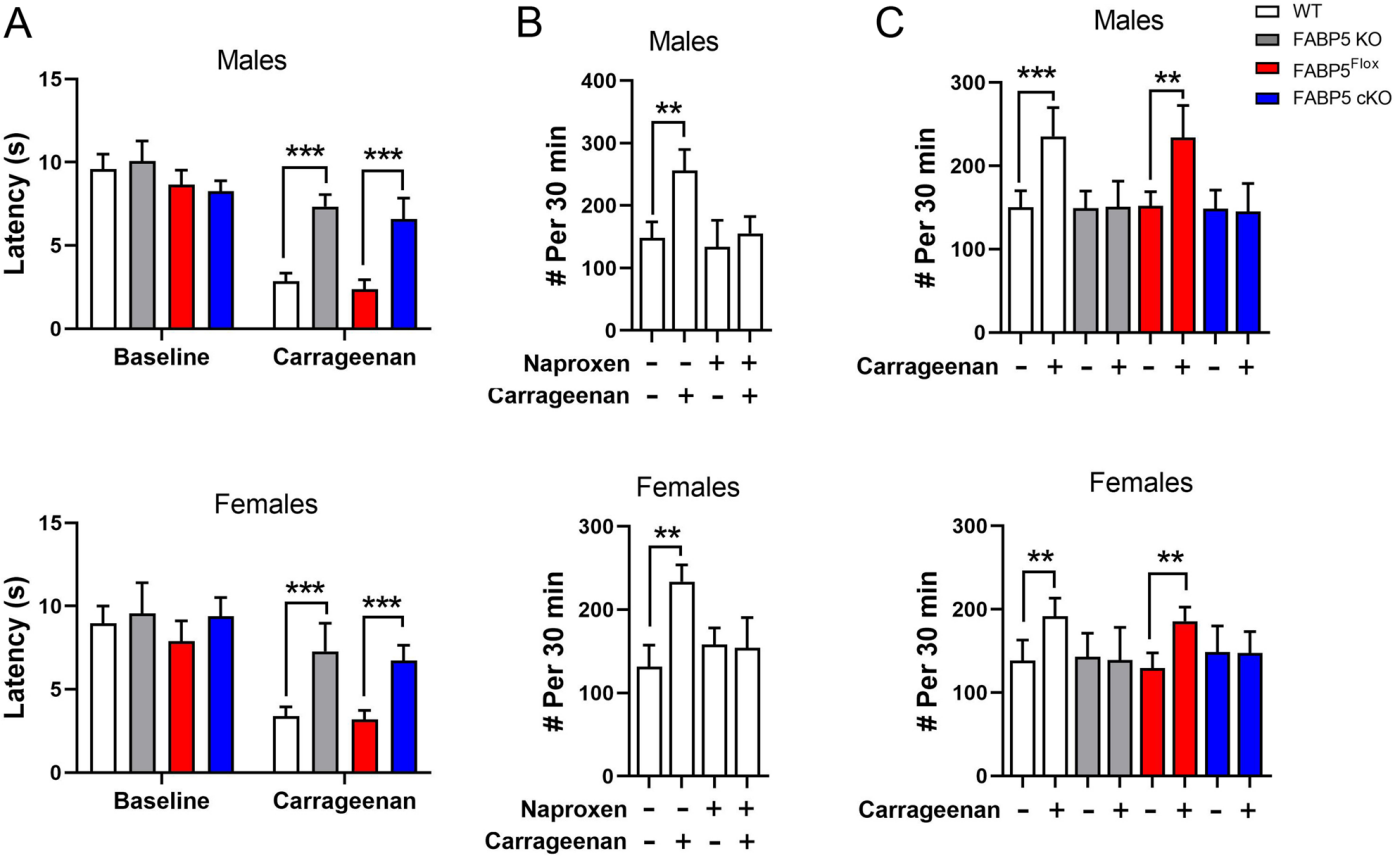
Deletion of FABP5 in nociceptors suppresses inflammatory hyperalgesia. (**A**) Thermal withdrawal latencies in male and female WT, FABP5 KO, FABP5^Flox^, and FABP5 cKO mice at baseline and 4 h after intraplantar carrageenan injection. ***p < 0.001 calculated using one-way ANOVA followed by Tukey post-hoc test (n = 6). (**B**) Behavioral spectrometer analysis of still behavior at baseline and after carrageenan injection in WT male and female mice administered naproxen (30 mg/kg, i.p.) or vehicle. **p < 0.01 calculated using paired *t* test (n = 6). (**C**) Still behavior in male and female WT, FABP5 KO, FABP5^Flox^, and FABP5 cKO mice at baseline and 4 h after carrageenan injection. **p < 0.01; ***p < 0.001 calculated using paired *t* test (n = 6).

We previously reported that pharmacological or genetic inhibition of FABP5 produces antinociceptive effects in models of inflammatory pain^43–45,48,49^. Consequently, we assessed inflammatory hyperalgesia in mice receiving an intraplantar injection of carrageenan^49^. As expected, WT and FABP5^Flox^ mice developed thermal hyperalgesia while paw withdrawal latencies were significantly higher in global FABP5 KO and FABP5 cKO mice (Fig. 2A)^50^, providing evidence that FABP5 deletion in sensory neurons produces antinociceptive effects. Comparable effects were observed in female mice (Fig. 2B), indicating that the function of FABP5 in pain modulation extends to both sexes. We additionally employed the behavioral spectrometer as a non-reflexive readout of inflammatory pain. The behavioral spectrometer quantifies diverse behavioral patterns in mice, a subset of which are pain-related and can be rescued by analgesics^51^. Of the behavioral patterns assessed, we found that carrageenan administration to male and female WT mice resulted in an increase in “still” behavior while the remaining parameters were unchanged (Fig. 2B, Supplementary Fig. 2), suggesting that mice remained more stationary in the setting of acute inflammatory pain. Administration of naproxen restored “still” patterns to pre-carrageenan baselines, confirming that these effects were pain-specific (Fig. 2B). Notably, while carrageenan administration increased “still” behavior in male and female FABP5^Flox^ mice, it failed to do so in FABP5 KO and FABP5 cKO mice (Fig. 2c).

### Contribution of CB1 and PPARα to pain hypersensitivity in FABP5 cKO mice

**Figure 3:**
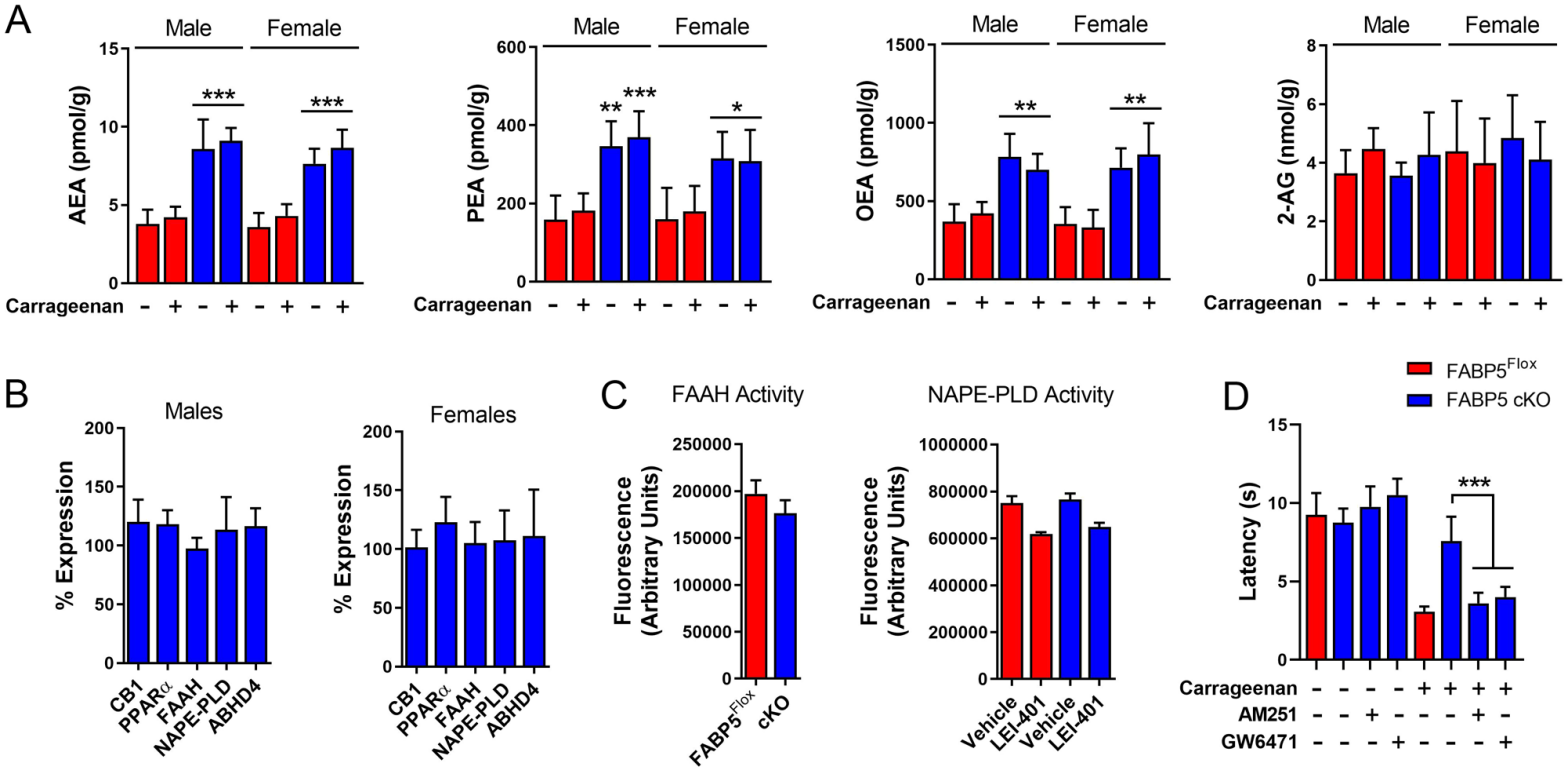
CB1 and PPARα mediate the antinociceptive effects in FABP5 cKO mice. (**A**) Levels of AEA, PEA, OEA, and 2-AG in DRGs of male and female FABP5^Flox^ and FABP5 cKO mice at baseline and 4 h after intraplantar carrageenan injection. **p < 0.01; ***p < 0.001 vs WT and FABP5^Flox^ calculated using two-way ANOVA followed by Bonferroni post-hoc test (n = 5). (**B**) Percent expression of CB1, PPARα, FAAH, NAPE-PLD, and ABHD4 in DRGs of male and female FABP5 cKO mice relative to FABP5^Flox^ controls. (**C**) FAAH and NAPE-PLD activity in DRGs. Left: DRGs were incubated with the selective FAAH substrate AMC-AEA (100 µM). Right: DRGs were incubated with the phospholipase A1 substrate PED-A1 (10 µM) in the presence or absence of the selective NAPE-PLD inhibitor LEI-401 (30 µM) (n = 3). (**D**) Thermal withdrawal latencies in FABP5 cKO mice receiving vehicle, AM251 (3 mg/kg, i.p.), or GW6471 (4 mg/kg, i.p.). ***p < 0.001 calculated using one-way ANOVA followed by Dunnett’s post-hoc test (n = 6).

To characterize the mechanisms underlying pain modulation by FABP5, we first quantified the levels of AEA, 2-AG, PEA, and OEA in DRGs of FABP5^Flox^ and FABP5 cKO mice. Compared to FABP5^Flox^ mice, AEA, PEA, and OEA levels were elevated in DRGs obtained from FABP5 cKO mice while 2-AG levels remained unchanged, and no sex-specific differences were noted (Fig. 3A). Carrageenan administration did not alter AEA, PEA, or OEA levels in FABP5 cKO mice (Fig. 3A). The expression of FAAH and the N-acylethanolamine biosynthetic enzyme NAPE-PLD were comparable between the genotypes and sexes (Fig. 3B). To validate these results, we employed the FAAH substrate AMC-AEA (Supplementary Fig. 3) to assess FAAH activity in DRGs of both genotypes, which revealed comparable activity levels (Fig. 3C). Similarly, we observed comparable hydrolysis of the phospholipase A1 substrate PED-A1 in DRGs of these mice (Fig. 3C). Since PED-A1 is not specific for NAPE-PLD, we treated DRGs with the selective NAPE-PLD inhibitor LEI-401 to identify the enzymatic activity corresponding to NAPE-PLD (Supplementary Fig. 3). Our results revealed comparable PED-A1 hydrolysis in DRGs of both genotypes treated with vehicle or LEI-401 (Fig. 3C). Taken together, these results indicate that the elevations in N-acylethanolamine levels observed in FABP5 cKO mice stem from attenuated transport and metabolism rather than alterations in biosynthetic or catabolic enzymes. Since no sex-specific differences were observed in any of the parameters assessed, we employed mixed cohorts of male and female mice for all subsequent experiments. To determine whether activation of CB1 or PPARα mediates the antinociceptive effects observed in FABP5 cKO mice, we treated mice with the CB1 antagonist AM251 (3 mg/kg, i.p.) or the PPARα antagonists GW6471 (4 mg/kg, i.p.). Our results indicate that AM251 and GW6471 restored thermal pain hypersensitivity in FABP5 cKO mice to levels observed in FABP5^Flox^ mice (Fig. 3D), providing direct evidence that deletion of FABP5 in sensory neurons reduces inflammatory hyperalgesia via CB1 and PPARα receptors.

### FABP5 inhibition reduces inflammation- and TRPV1-evoked CGRP release

**Figure 4:**
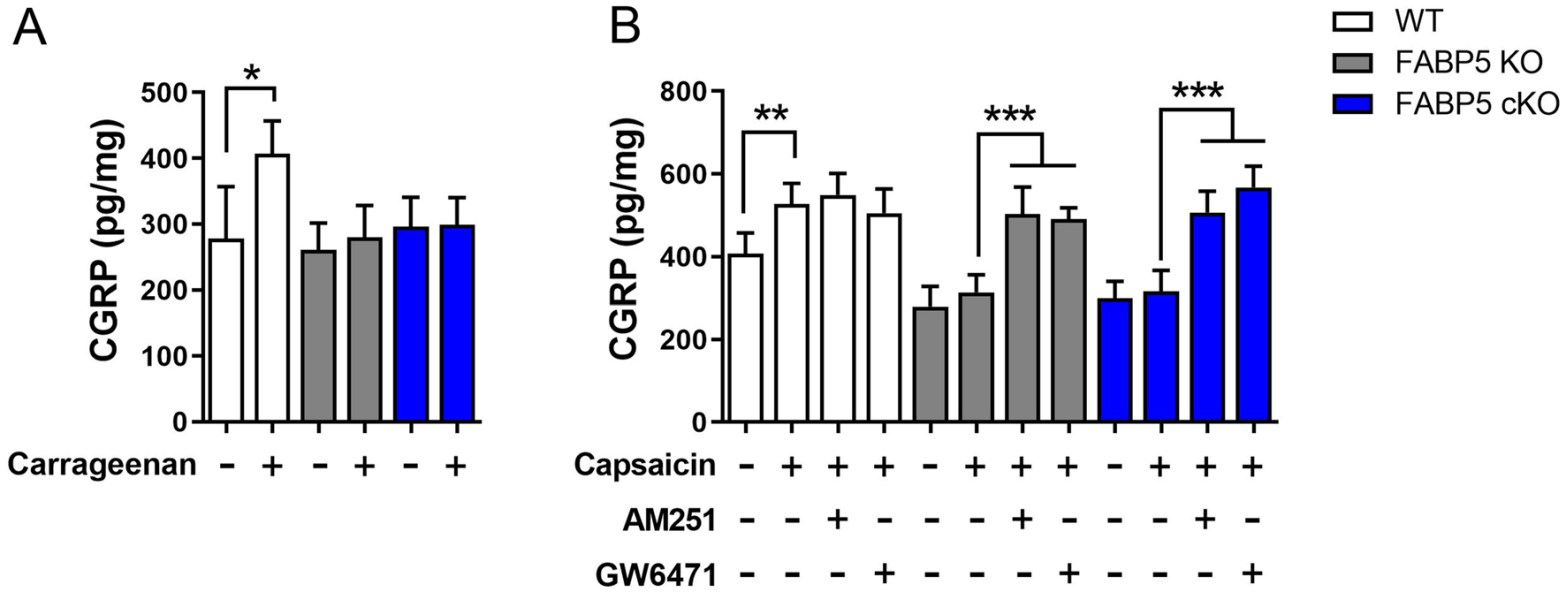
FABP5 deletion attenuates carrageenan-induced and TRPV1-evoked spinal CGRP release. (**A**) WT, FABP5 KO, and FABP5 cKO mice received intraplantar injections of vehicle or carrageenan and lumbar spinal cords were obtained 4 h later. The sections were incubated for 30 min and CGRP release was subsequently quantified. *p < 0.05 calculated using unpaired *t* test (n = 5). (**B**) WT, FABP5 KO, and FABP5 cKO mice were administered vehicle, 3 mg/kg AM251, or 4 mg/kg GW6471 prior to intraplantar injections of carrageenan. Lumbar spinal cord sections were collected 4 h later and incubated with vehicle followed by 1 µM capsaicin in the presence or absence of 3 µM AM251 or 10 µM GW6471 for 30 min. CGRP release was quantified prior to and after the addition of capsaicin. **p < 0.01; ***p < 0.001 calculated using one-way ANOVA followed by Dunnett’s post-hoc test (n = 5).

Inflammatory hyperalgesia is characterized by exacerbated release of CGRP from sensory neuron terminals into the dorsal horn of the spinal cord^29,30,52^. CGRP levels are elevated in rodents and humans experiencing ongoing pain and are reduced by analgesics^29,30^, thereby serving as a neurochemical proxy for nociceptor sensitization/output. We previously reported that the carrageenan-induced increase in spinal CGRP levels is blunted following global FABP5 deletion^49^. Consistent with these results, CGRP release was suppressed in both FABP5 KO and cKO mice following carrageenan challenge (Fig. 4A), confirming that FABP5 deletion in nociceptors recapitulates the effects observed in global FABP5 KO mice.

TRPV1 is extensively co-expressed with CGRP and FABP5 in sensory neurons^45,53,54^ and carrageenan administration sensitizes TRPV1, leading to exacerbated CGRP release^49^. Consistent with this notion, while capsaicin-evoked CGRP release was enhanced in WT mice, its release was blunted in spinal cords of FABP5 KO and FABP5 cKO mice (Fig. 4B), indicating that FABP5 deletion in sensory neurons leads to attenuated TRPV1 sensitization. Administration of AM251 or GW6471 to FABP5 KO or cKO mice restored CGRP release to levels comparable to WT mice (Fig. 4B), confirming that CB1 and PPARα activation underlies these effects.

### FABP5 deletion blunts TRPV1 sensitization via CB1 and PPARα

**Figure 5:**
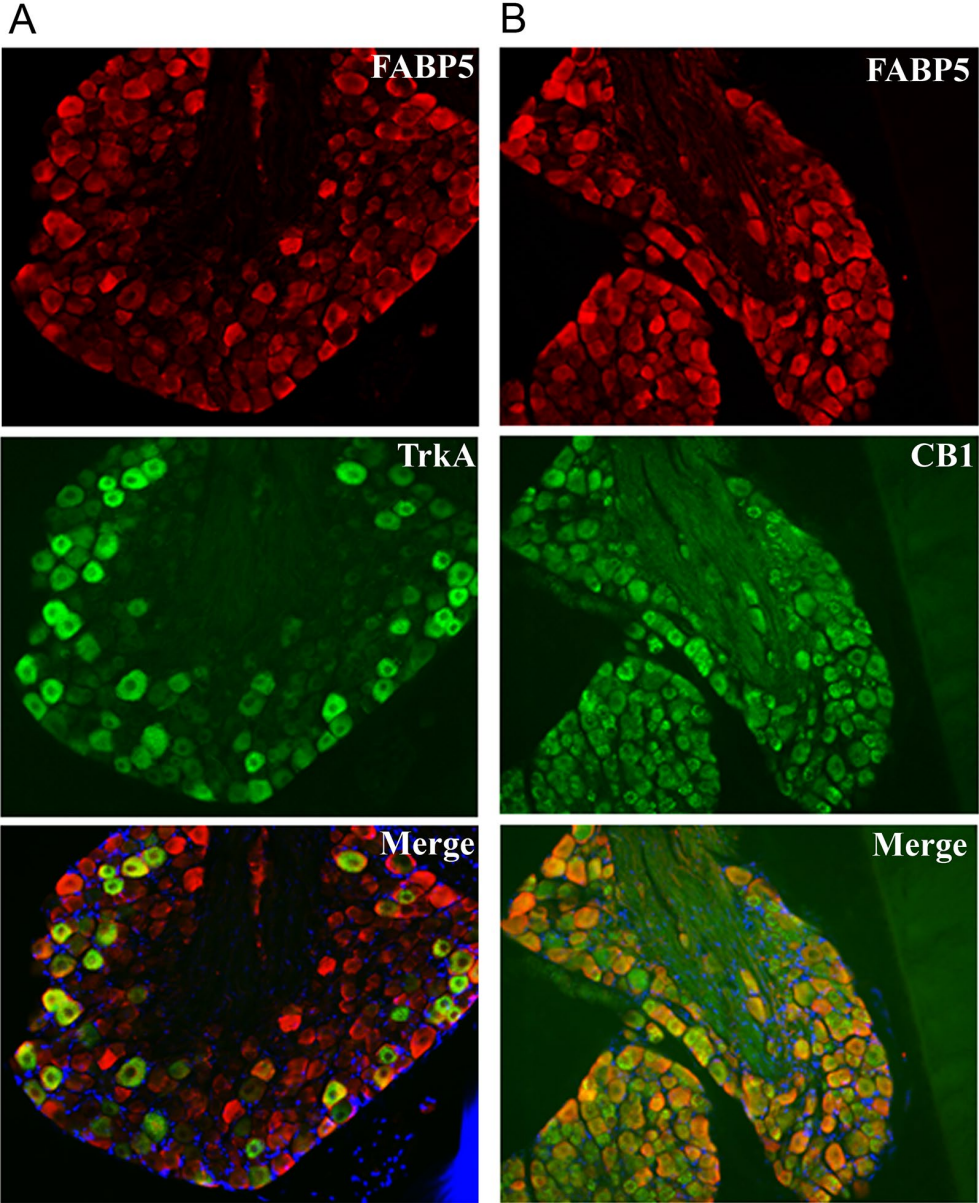
Co-expression of FABP5 with (**A**) TrkA and (**B**) CB1 in DRG neurons. Representative images of FABP5, CB1, and TrkA distribution in L4-L5 DRGs. DAPI is shown in blue.

**Figure 6:**
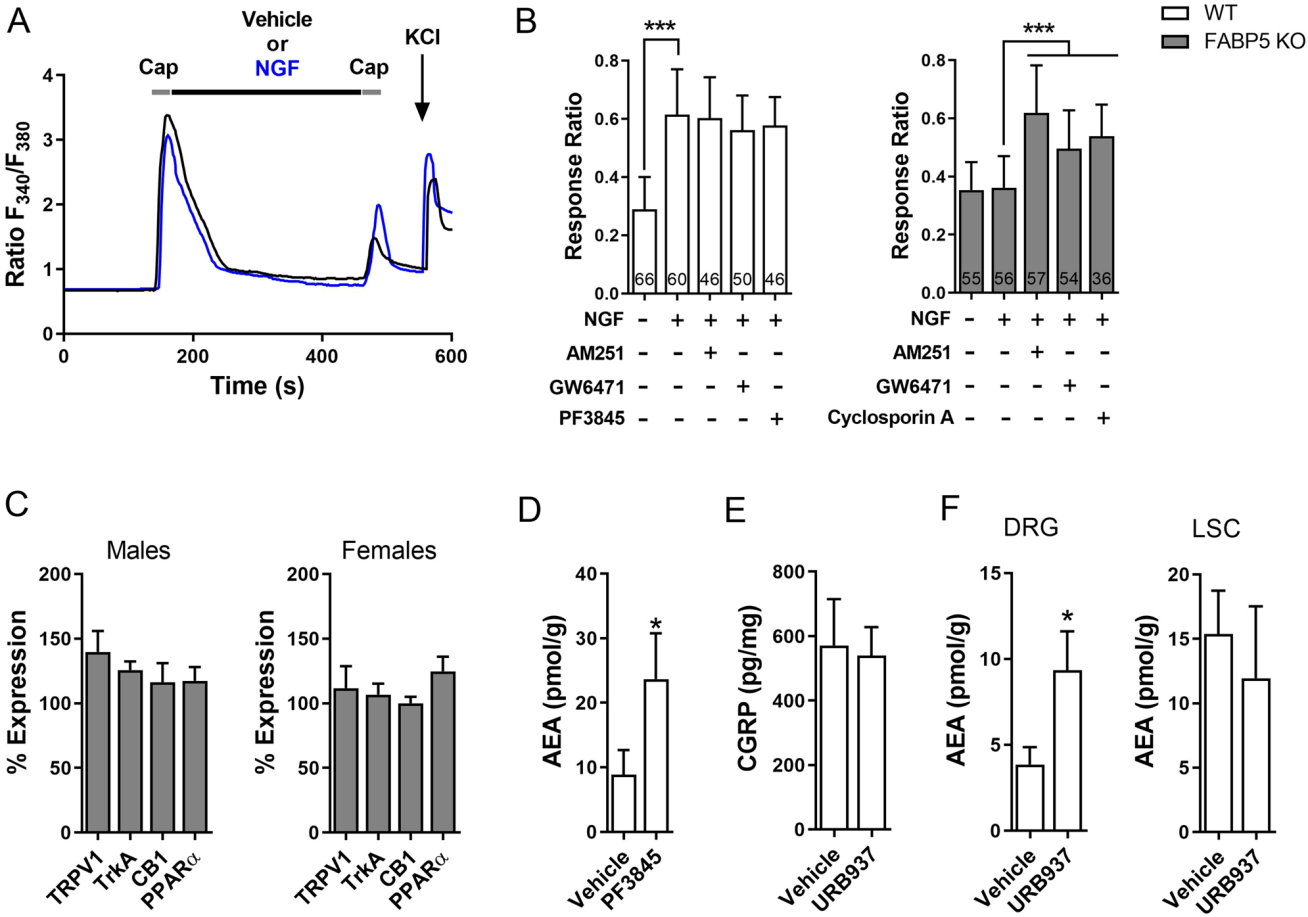
FABP5 deletion blunts NGF-mediated TRPV1 sensitization via CB1 and PPARα. (**A**) Design of calcium imaging experiment and representative calcium traces in WT and FABP5 KO DRGs incubated with NGF and capsaicin. DRG neurons received two 30 s pulses of 100 nM capsaicin (Cap, gray bars) interspersed with a 5 min incubation of vehicle (black trace) or 100 ng/ml NGF (blue trace). KCl was employed to confirm neuronal identity. (**B**) Capsaicin response ratio (2nd/1st peak) in WT (white bars) and FABP5 KO (gray bars) DRGs incubated with vehicle or NGF in the presence or absence of 3 µM AM251, 10 µM GW6471, or 200 nM cyclosporin A. The effect of FAAH inhibition upon capsaicin responses was evaluated by incubating WT DRGs with 1 µM PF3845 for 2 h prior to capsaicin application. ***p < 0.001 calculated using one-way ANOVA followed by Dunnett’s post-hoc test. (**C**) qPCR analysis of relative expression levels of TRPV1, TrkA, CB1, and PPARα in male and female FABP5 KO DRGs relative to WT controls (n = 3). (**D**) Levels of AEA in WT DRGs incubated for 2 h with vehicle or 1 µM PF3845. *p < 0.05 calculated using unpaired *t* test (n = 3). (**E**) Capsaicin-evoked CGRP release in WT mice administered vehicle or URB937 (1 mg/kg, i.p.). Mice received URB937 1 h prior to intraplantar carrageenan administration and spinal CGRP release elicited by 1 µM capsaicin was examined 4 h later (n = 5). (**F**) AEA levels in DRGs and lumbar spinal cords (LSC) of mice injected with vehicle or URB937 (1 mg/kg, i.p.). *p < 0.05 calculated using unpaired *t* test (n = 3).

Carrageenan-evoked hyperalgesia is dependent upon nerve growth factor (NGF) release, which plays an obligate role in TRPV1 sensitization^55–59^. NGF activates the TrkA receptor that is co-expressed with TRPV1 in numerous sensory neurons^60^. We confirmed that DRG neurons co-express FABP5 and TrkA (Fig. 5A) and subsequently employed calcium imaging to determine the impact of FABP5 deletion upon TRPV1 sensitization by NGF. TRPV1 undergoes rapid tachyphylaxis following repeated capsaicin application^61^ that can be largely restored by mediators that sensitize TRPV1 including NGF (Fig. 6A). Our results revealed that while NGF sensitized TRPV1 in WT DRGs, it failed to do so in DRGs obtained from FABP5 KO mice (Fig. 6B). Comparable TRPV1 and TrkA expression was observed between the genotypes (Fig. 6C), confirming that these results do not stem from altered TRPV1 or TrkA expression. Application of the CB1 antagonist AM251 restored the ability of NGF to sensitize TRPV1 in FABP5 KO DRGs (Fig. 6B) and is consistent with the extensive co-expression between FABP5 and CB1 (Fig. 5B). The PPARα antagonist GW6471 was likewise effective in restoring the ability of NGF to sensitize TRPV1 (Fig. 6B). CB1 and PPARα expression was comparable between the genotypes (Fig. 6C). Mechanistically, exogenously applied CB1 agonists suppress TRPV1 sensitization via calcineurin^62^. Consistent with these observations, the calcineurin inhibitor cyclosporin A largely restored TRPV1 sensitization in FABP5 KO DRGs (Fig. 6B), indicating that a common mechanism is shared between exogenous and endogenous CB1 agonists. Collectively, these data provide evidence that FABP5 deletion promotes CB1 and PPARα activation to blunt NGF-mediated TRPV1 sensitization.

**Figure 7:**
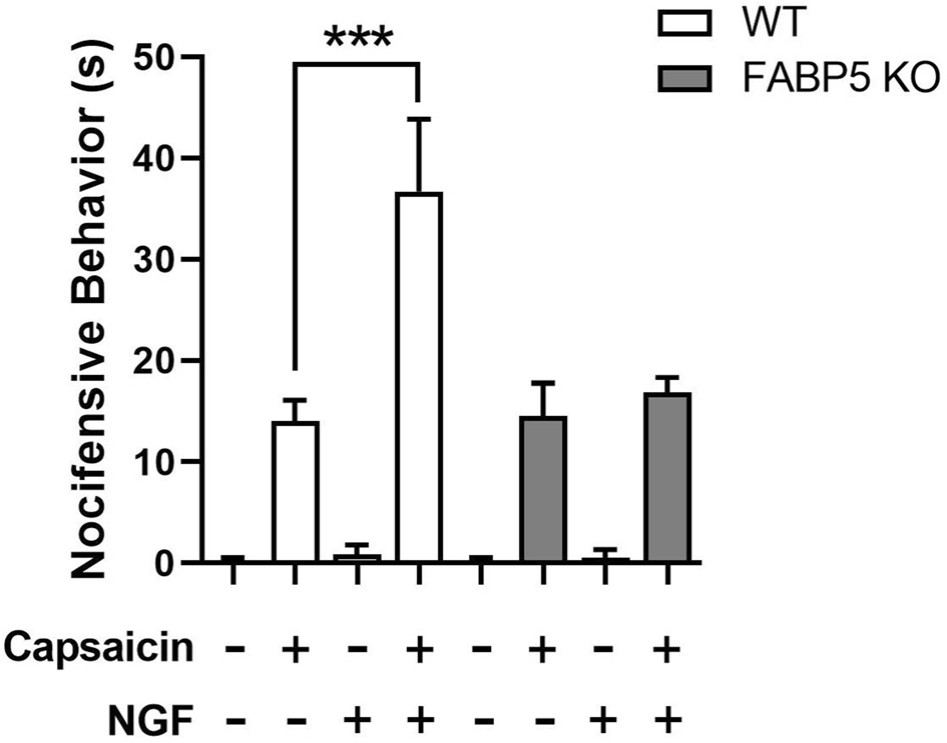
Capsaicin-induced nocifensive behavior in WT and FABP5 cKO mice. Mice received an intraplantar injection of vehicle or 200 ng NGF followed by vehicle or 1 µg capsaicin. The duration of nocifensive behavior defined as paw licking or biting was subsequently scored for 5 min. *p < 0.05 calculated using unpaired *t* test (n = 6).

To determine whether these effects are observed in vivo, we assessed capsaicin-evoked nocifensive (paw licking/biting) behaviors in WT and FABP5 KO mice pre-treated with vehicle or NGF. Intraplantar capsaicin induced comparable nocifensive behaviors in WT and FABP5 KO mice at baseline (Fig. 7). In contrast, while administration of NGF potentiated capsaicin-evoked nocifensive behaviors in WT mice, it failed to do so in mice lacking FABP5 (Fig. 7). Administration of vehicle or NGF alone did not elicit a robust behavioral response. Collectively, our results provide direct evidence that FABP5 is essential for NGF-mediated TRPV1 sensitization in vivo.

### FAAH inhibition produces distinct effects upon TRPV1

Based on the role of FABP5 as an intracellular carrier that delivers AEA to FAAH^39^, inhibition of FAAH is expected to phenocopy the effects of FABP5 upon TRPV1 sensitization. However, FAAH inhibition was previously reported to enhance TRPV1 activation in DRG neurons while intraplantar capsaicin administration exacerbated nocifensive behaviors in FAAH KO mice^7,27^. These results suggest that blockade of FAAH and FABP5 may produce non-overlapping and possibly opposing effects upon TRPV1 sensitization. We explored this possibility and found that application of the selective FAAH inhibitor PF3845 failed to blunt NGF-mediated TRPV1 sensitization despite elevating AEA levels in DRGs (Fig. 6B,D). Moreover, administration of the peripherally-restricted FAAH inhibitor URB937 (1 mg/kg, i.p.)^19^ did not suppress capsaicin-evoked CGRP release (Fig. 6E,F). Taken together, these results provide further evidence that while inhibition of both FABP5 and FAAH results in elevated AEA levels, distinct effects upon TRPV1 sensitization are observed.

## Discussion

Augmentation of endocannabinoid signaling represents an attractive strategy to treat pain. Initial translational efforts to harness the therapeutic potential of the endocannabinoid system were dampened following the failure of a FAAH inhibitor in a clinical trial of osteoarthritis pain^20^. A mechanism(s) underlying this seeming lack of efficacy has yet to emerge. While systemic elevations in AEA levels produce antinociceptive effects in most preclinical models of pain, inflammatory mediators can convert AEA into a potent TRPV1 agonist in nociceptive sensory neurons while TRPV1-mediated nocifensive responses are observed in mice lacking FAAH^24,27^. Therefore, it is conceivable that the dual function of AEA as a CB1 and TRPV1 agonist may contribute at least in part to the observed lack of efficacy of the FAAH inhibitor. However, the molecular targets engaged by AEA in nociceptors in vivo have not been established and the goal of the current work was to determine whether FABP5 deletion and ensuing AEA elevation leads to CB1-mediated antinociceptive effects or TRPV1-mediated pro-nociceptive effects.

Previous efforts to characterize the role of the peripheral endocannabinoid system in pain modulation have yielded important insights at the behavioral level. Administration of peripherally-restricted FAAH inhibitors resulted in CB1- and/or PPARα-mediated antinociceptive effects^19^, providing strong evidence that enhancement of the peripheral AEA and PEA tone is sufficient to modulate pain. Moreover, deletion of CB1 receptors from nociceptors abolished the antinociceptive effects of exogenous CB1 agonists^4^, further positioning CB1 receptors in sensory neurons as key determinants of cannabinoid analgesia. Our present results demonstrate that FABP5 mediates AEA transport in DRGs as its deletion elevates AEA levels, consistent with the high degree of co-expression between FABP5 and FAAH^45^. Our combined histological and behavioral data reveal that FABP5 is expressed in CB1^+^ sensory neurons while its ablation augments AEA levels to trigger antinociceptive effects mediated by CB1 receptors. The selective increase in AEA but not 2-AG levels likely suggests that monoacylglycerol lipase may maintain access to 2-AG on the plasma membrane while bypassing the need for cytosolic transport by FABP5. Alternatively, the possibility that other proteins contribute to cytosolic 2-AG trafficking cannot be excluded at this time.

The molecular mechanisms underlying pain modulation by endocannabinoids have been partially elucidated with TRPV1 emerging as a major target of interest given its prominent role in pain. It was previously demonstrated that application of exogenous CB1 agonists disrupts TRPV1 sensitization in DRG neurons in vitro through a calcineurin-dependent mechanism^60,62,63^. Employing capsaicin-evoked CGRP release and NGF-mediated TRPV1 sensitization as readouts, our data revealed that FABP5 ablation elevates AEA levels and blunts TRPV1 sensitization via a CB1- and calcineurin-mediated mechanism. TRPV1 sensitization was also restored following antagonism of PPARα, consistent with a report demonstrating that PPARα physically interacts with TRPV1 and its activation desensitizes the channel^64^. Notably, these data support our previous findings that inhibition of peripherally-expressed FABP5 produces antinociceptive effects via CB1 and PPARα^45^. Unexpectedly, the inability of FAAH inhibition to block TRPV1 sensitization coupled with previous reports that FAAH inhibition promotes TRPV1 activation and pro-nociceptive effects^24,27,34–38^ highlights divergent roles of FABP5 and FAAH in controlling TRPV1 function.

There are several potential mechanisms that could underlie these contrasting effects. It is possible that FABP5 transports AEA and/or other FAAH substrates to TRPV1 for activation. Consequently, FABP5 deletion may compromise ligand delivery to TRPV1 while this function is preserved following FAAH inhibition leading to TRPV1 engagement. Additionally, it is possible that FABP5 and FAAH regulate the transport and metabolism of non-overlapping pools of TRPV1 ligands. For example, N-acyl taurines are TRPV1 agonists that are highly elevated following FAAH inhibition^26^ and could in principle counteract AEA- and CB1-mediated antinociceptive effects. If N-acyl taurines are not FABP5 ligands and their levels unaltered following FABP5 inhibition, this could permit AEA-mediated antinociceptive effects to predominate. Regardless of the mechanism(s) involved, it is tempting to speculate that the inability of FAAH inhibition to temper TRPV1 sensitization may contribute at least in part to the lack of efficacy of a FAAH inhibitor in a clinical trial for pain^20^. Collectively, our results establish a key role for FABP5 in shaping endocannabinoid and N-acylethanolamine signaling in nociceptors with FABP5 emerging as a novel modulator of TRPV1 sensitization. The contrasting effects between FABP5 and FAAH coupled with previously established anti-inflammatory effects observed following FABP5 inhibition position FABP5 as a promising target for the development of analgesics^49^.

## Methods

### Compounds

GW6471, AM251, URB937, PF3845, AMC-AEA, LEI-401, PEA, d_4_-PEA, OEA, d_2_-OEA, 2-AG, and d_5_-2-AG were purchased from Cayman Chemical (Ann Arbor, MA). NGF was from Gibco (Waltham. MA). AEA and d_4_-AEA were from R&D Systems (Minneapolis, MN). Capsaicin was from Sigma-Aldrich (St. Louis, MO), Fura-2AM and PED-A1 were from Thermo Fisher (Waltham, MA) and Cyclosporin A was from TCI (Portland, OR).

### Generation of FABP5^Flox^ and FABP5 cKO mice

Mice carrying LoxP-flanked exons 2 and 3 were generated via standard gene-targeting techniques by Cyagen. A 17.5 Kb targeting vector containing a diphtheria toxin (DTA) cassette, neomycin cassette flanked by Frt sites, and exons 2–3 flanked by LoxP sites was electroporated into C57Bl/6 ES cells, which were subsequently selected for blastocyst microinjections. Founders were confirmed as germline-transmitted via crossbreeding with Flp-deleter mice. The F1 progeny were subsequently backcrossed to C57Bl/6 mice and then crossed with Trpv1-Cre mice (Jackson Labs #017769) to ablate FABP5 in TRPV1^+^ nociceptors. Control C57Bl/6J mice were from Jackson Labs.

### Hargreaves

Thermal hyperalgesia was assessed as previously reported^43^. Briefly, inflammation was induced by a unilateral, intraplantar injection of 1% carrageenan (20 µL in saline). The average latency to withdraw the paw was measured when a focused beam of radiant heat was applied to the mid plantar surface using a Hargreaves plantar apparatus (Ugo Basile). Mice were acclimated to the experimental room and Hargreaves for 2 days prior to baseline testing and at least 1 h prior to testing.

### Behavioral spectrometer

Mice were acclimated to the Behavioral Sequencer box (Behavioral Instruments Inc, New Jersey) for 2 days prior to testing^51^. Mice were allowed to move around freely within the 40 cm by 40 cm arena for 30 min during testing. Using a combination of cameras, infrared sensors and accelerometers embedded in the floor, the frequency and duration of predetermined behaviors were analyzed with Viewer 3 software (BiObserve Inc, St Augustin, Germany). Mice were placed in the box 4 h after carrageenan or saline injection.

### Capsaicin-induced nocifensive behavior

Mice were acclimated for two days to a transparent Plexiglass enclosure (8″ × 8″) and allowed to move freely for 20 min. On the day of testing, the mice received an intraplantar injection of 200 ng NGF (Gibco, Waltham, Massachusetts) or vehicle (saline, 10 µl) followed by 1 µg capsaicin (Sigma-Aldrich, St Louis, MO) or vehicle (5% ethanol in saline, 10 µl) 30 min later. The duration of nocifensive behaviors (paw licking and/or biting) was assessed for 5 min immediately following the injections.

### Immunofluorescence

Mice were anesthetized with isoflurane and transcardially perfused with saline followed by 4% paraformaldehyde (PFA) in 0.1 M phosphate buffered saline (PBS), pH 7.4. DRGs were dissected out from the spinal column and immersed in the same fixative overnight at 4ºC. The next day the tissue was immersed in 30% sucrose in 0.1 M PB for cryoprotection. The DRGs were embedded in a gelatin-albumin mixture (3% gelatin, 30% egg albumin in dH_2_O) and frozen using a liquid nitrogen-chilled isopentane bath. Cryostat sections 16 µm in thick were thaw-mounted on gelatin/chromium-coated glass slides, air dried, and stored in a frost-free freezer at − 20 °C until further processing. For immunofluorescence staining slides were thawed at room temperature for 10 min. Sections were fixed onto the slides with 4% PFA for 3 min, rinsed with PBS three times for 10 min each, and incubated for 30 min in PBS containing 5% normal donkey serum (NDS). Slides were incubated 24–48 h at 4 °C with a mixture of primary antibodies (Table 1) diluted in PBS with 0.3% Triton X-100 and 5% NDS. Following primary antibody incubation, the sections were again washed three times for 10 min each in PBS followed by 30 min in 5% NDS in PBS. The slides were incubated for 1 h at 37 °C with a mixture of secondary antibodies conjugated to either Alexa 488 or 594 diluted in PBS with 0.3% Triton X-100 and 5% NDS. Slides were subsequently washed three times with PBS for 10 min each and immediately mounted with Vectashield Plus® antifade mounting medium with DAPI (Vector Labs Burlingame, CA, Cat# H-2000) and stored at 4 °C in the dark. Fluorescent immunoreactivity in cells was observed with a Zeiss Axioplan 2 epifluorescent microscope. Images were obtained using Zeiss AxioCam HRm monochrome digital camera, and AxioVision Rel. 4.6 microscope software. Images were only adjusted for brightness and contrast.

**Table 1:** Primary antibodies used in the current manuscript.

Antibody	Dilution	Supplier	Cat #	RRID
FABP5	1:500	BioVendor	RD181060100	AB_344491
FABP5	1:600	R&D Systems	AF1476	AB_2293656
TRPV1	1:600	EMD Millipore	AB5566	AB_91901
CB1	1:500	Abcam	Ab172970	AB_2692328
TrkA	1:200	R&D Systems	AF1056	AB_2283049

### qPCR

RNA was extracted using the RNeasy mini kit (Qiagen) followed by cDNA synthesis using the SuperScript III First Strand synthesis system (ThermoFisher). qPCR was performed with PowerUp SYBR green (ThermoFisher) on a StepOnePlus instrument (Applied Biosystems). Quantification was performed using the 2^−ΔΔCt^ method with actin serving as the housekeeping gene. The following primers were used: FABP5: TGGTCCAGCACCAGCAATG and GACACACTCCACGATCATCTTC; CB1: AAGTCGATCTTAGACGGCCTT and TCCTAATTTGGATGCCATGTCTC; FAAH: CCCTGCTCCAACTGGTACAG and TCACAGTCAGTCAGATAGGAGG; NAPE-PLD: CTCCTGGACGACAACAAGGTTC and GCAAGGTCAAAAGGACCAAAC; ABHD4: TTCCCCTACGACCAACTGAC and CGAAGAACAGCCAGTGGATT; PPARα: GCCTGTCTGTCGGGATGT and GGCTTCGTGGATTCTCTTG; TRPV1: AAGGCTCTATGATCGCAGGA and CAGATTGAGCATGGCTTTGA; TrkA: GGTCTTTCTCGCTGAGTGCTAC and GCTGAAAGTCCTGCCGAGCATT; Actin: GACGGCCAGGTCATCACTAT and CGGATGTCAACGTCACACTT.

### CGRP

CGRP release was performed as previously described^49^. Mice were administered vehicle, AM251 (1 mg/kg), GW6471 (4 mg/kg), or URB937 (1 mg/kg) via the i.p. route and received bilateral intraplantar injections of 1% λ-carrageenan 30 min later. After 4 h, L4-L5 lumbar spinal sections were incubated in EC1A buffer (10 mM HEPES, 13 mM glucose, 151 mM NaCl, 2.5 mM KCl, 1 mM MgCl_2_, 2 mM CaCl_2_, pH 7.4) containing vehicle (0.1% DMSO), AM251 (3 µM), or GW6471 (10 µM) for 30 min at 37 °C. Media were collected and the samples were subsequently incubated with 1 µM capsaicin in the presence of the antagonists or URB937 for an additional 30 min. The media were again collected and CGRP release quantified using a CGRP ELISA kit (Bertin Bioreagent A05482-CGRP) and normalized to protein concentration.

### FAAH and NAPE-PLD activity assays

To quantify FAAH activity, DRG homogenates (100 µg) were incubated with 100 µM AMC-AEA in Tris buffer (50 mM, pH 8) for 60 min at 37 °C. AMC-AEA hydrolysis was quantified on a SpectraMax i3x plate reader (Molecular Dynamics) using excitation and emission wavelengths of 360 and 465 nm, respectively. For NAPE-PLD activity measurements, DRG homogenates were incubated with 10 µM PED-A1 in the presence or absence of the NAPE-PLD inhibitor LEI-401 (30 µM) in Tris buffer for 60 min at 37 °C. PED-A1 hydrolysis was quantified using excitation and emission wavelengths of 488 and 530 nm, respectively. Negative control reactions consisted of substrates incubated in Tris buffer alone and were subtracted from all activity data.

### Calcium imaging

Mice were decapitated while under deep anesthesia with isoflurane. The lumbar segment from L4-L5 were removed and placed in a cold Ca^2^^+^, Mg^2+^ free (CMF) Hank’s solution (Gibco, Waltham, MA) containing 137 mM NaCl, 5.3 mM KCL, 0.33 mM Na_2_HPO_4_, 0.44 mM KH_2_PO_4_, 5 mM HEPES, 5.5 mM glucose, pH 7.4. DRGs were removed, cut in half and incubated in Ca^2+^, Mg^2+^ free Hank’s solution containing 20 U/ml Papain (Worthington Biochemical, Lakewood, NJ) and 5 mM DL-cysteine (Sigma-Aldrich, St Louis, MO) for 20 min at 34 °C. DRGs were then incubated in 3 mg/ml collagenase (Type I, Sigma-Aldrich, St Louis, MO) and 4 mg/ml Dispase II (Boehringer Mannheim, IN) in CMF Hank’s solution for 20 min at 34 °C. DRGs were then washed with Leibovitz’s L-15 medium (Invitrogen, San Diego, CA) supplemented with 5 mM HEPES (Sigma-Aldrich, St Louis, MO) and 10% fetal calf serum solution (Gibco, Waltham, Massachusetts). After drawing off L-15 medium, 150 µl Dulbecco’s Modified Eagle medium (DMEM, Gibco, Waltham, Massachusetts) and 150 µl L-15 medium were added and DRGs were dispersed with mechanical trituration using fire-polished Pasteur pipettes with decreasing bore size. Cells were plated on glass coverslips coated with 100 mg/ml poly-D-lysine (Gibco, Waltham, Massachusetts) at 37 °C (in 5% CO_2_) for one hour. DMEM/L15 solution was replaced with Neurobasal supplemented with fetal bovine serum containing penicillin, streptomycin, and glutamine (Gibco, Waltham, Massachusetts) and cells incubated overnight at 37 °C in 5% CO_2_.

Dissociated DRG neurons were incubated with 5 µM fura-2AM in EC1A buffer for 30 min at 37 °C. DRG neurons loaded with fura-2AM were pulsed alternately with 340 and 380 nm wavelength illumination (Lambda XL, Sutter Instruments, Novato, CA). Images were acquired using the ORCA-Flash4.0 digital camera at a capture rate of 0.33 Hz. The fluorescence ratio for individual neurons was determined as the intensity of emission during 340 nm excitation (I340) divided by 380 nm emission (I380) and used as an indicator of change in cytoplasmic calcium. The I340/I380 ratio was calculated on a pixel-by-pixel basis using the MetaFluor software (Molecular Devices, Sunnyvale, CA, USA). NGF (2.5S murine, Gibco, Waltham, Massachusetts) at 100 ng/ml and capsaicin (Sigma-Aldrich, St Louis, MO) at 100 nM in EC1A were applied using an array of quartz fiber flow pipes (500 mm internal diameter) positioned about 1 mm away from DRGs. Drugs were added to Fura-2AM, EC1A, NGF and capsaicin solutions so that cells were continually bathed in the following concentrations: 10 µM GW6471, 1 µM AM251, 1 µM PF3845 (Cayman, Ann Arbor, MI), and 200 nM cyclosporin A (TCI, Portland, OR).

### Statistical analysis

Data are represented as mean ± SD. Thermal hyperalgesia was analyzed by One-Way ANOVA followed by Tukey post-hoc while behavioral spectrometer assays were analyzed via paired t-test. Changes in lipid levels were assessed via Two-Way ANOVA followed by Bonferroni post-hoc or unpaired t-test as appropriate. Carrageenan-evoked CGRP release assays were analyzed by unpaired t-test while capsaicin-evoked release was analyzed via One-Way ANOVA followed by Dunnett’s post-hoc or unpaired t-test as appropriate. Calcium imaging was examined via One-Way ANOVA followed by Dunnett’s post-hoc test. qPCR data were analyzed by comparing ΔCt values using an unpaired t-test.

### Ethics statement

All experiments were approved by the Stony Brook University Animal Care and Use Committee (#277150). The experiments met the United States Public Health Service Policy guidelines on Humane Care and Use of Laboratory Animals. Mice were housed in an AAALAC certified facility with ad libitum access to food and water, and lighting was maintained on a 12-h light/dark cycle. Data reporting in the manuscript follows the recommendations in the ARRIVE guideline. All experiments were performed on 8- to 12-week-old mice.

## Supplementary Information

Supplementary Information.

## Data Availability

Data will be available from the corresponding author upon reasonable request.
