# Supplementary material for: FABP5 deletion in nociceptors augments endocannabinoid signaling and suppresses TRPV1 sensitization and inflammatory pain
Source: Sci Rep. 2022 Jun 2;12:9241. doi: 10.1038/s41598-022-13284-0 (PMC9163147; doi:10.1038/s41598-022-13284-0)
Supplement: Supplementary file 1 — Supplementary Information. [file 41598_2022_13284_MOESM1_ESM.docx]

**
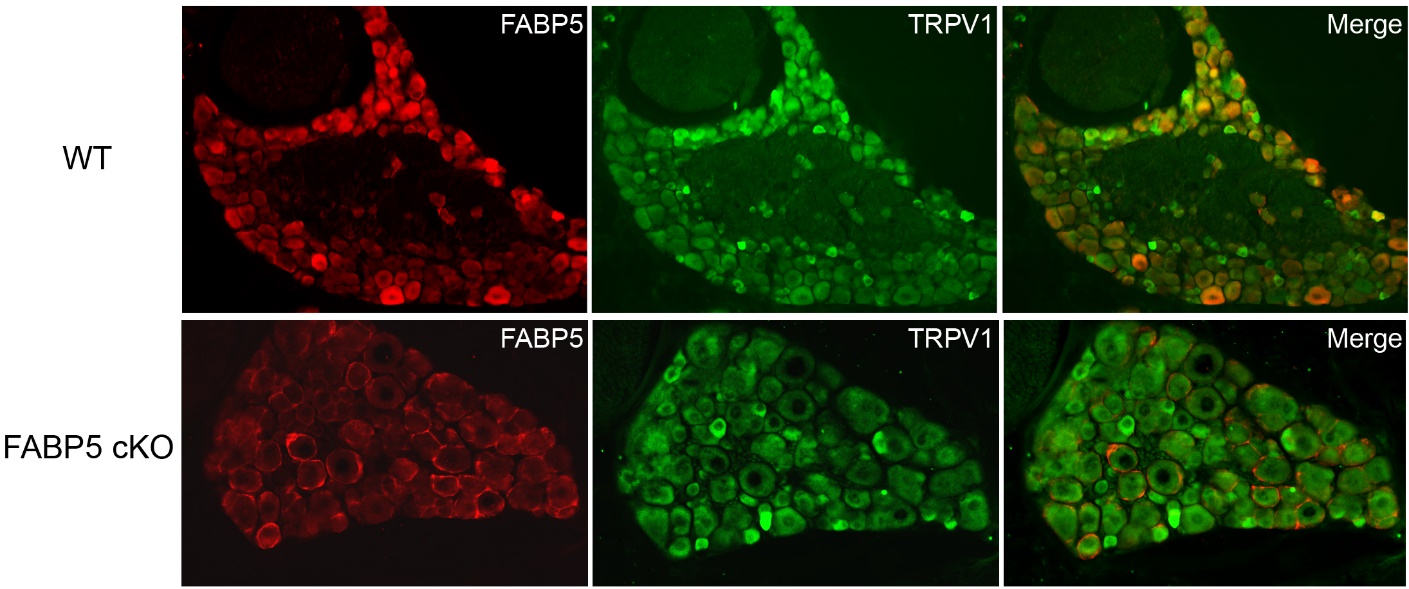
**

**Supplementary Figure 1.** FABP5 and TRPV1 immunolocalization in DRGs of WT and FABP5 cKO mice.

**
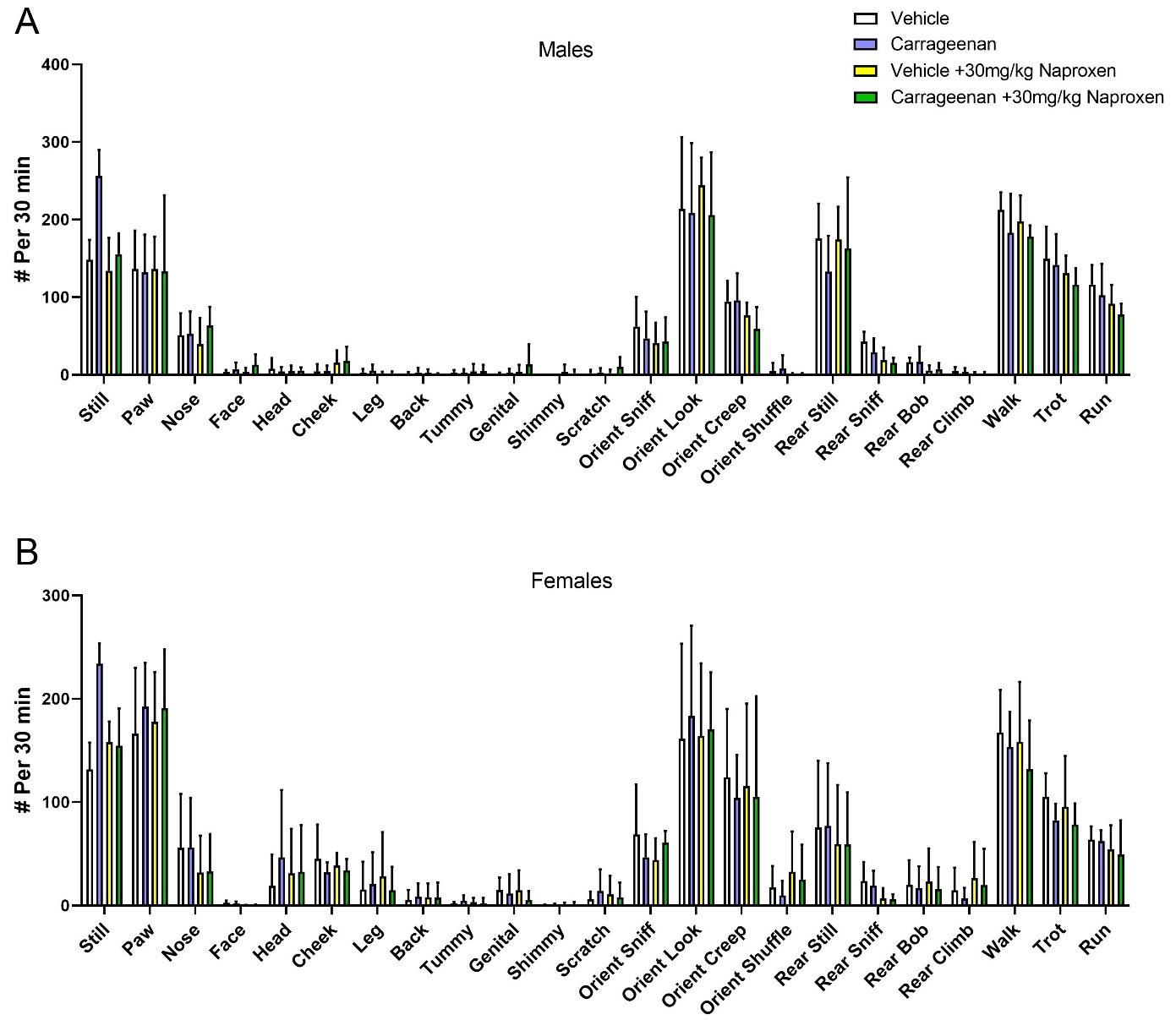
**

**Supplementary Figure 2.** Behavioral responses of WT male and female mice before and after carrageenan administration. Male (**a**) and female (**b**) mice were injected with vehicle or naproxen (30 mg/kg, i.p.) and behavioral responses were scored via the behavioral spectrometer. On the subsequent day, vehicle and naproxen administration was followed by injection of carrageenan (n = 6).


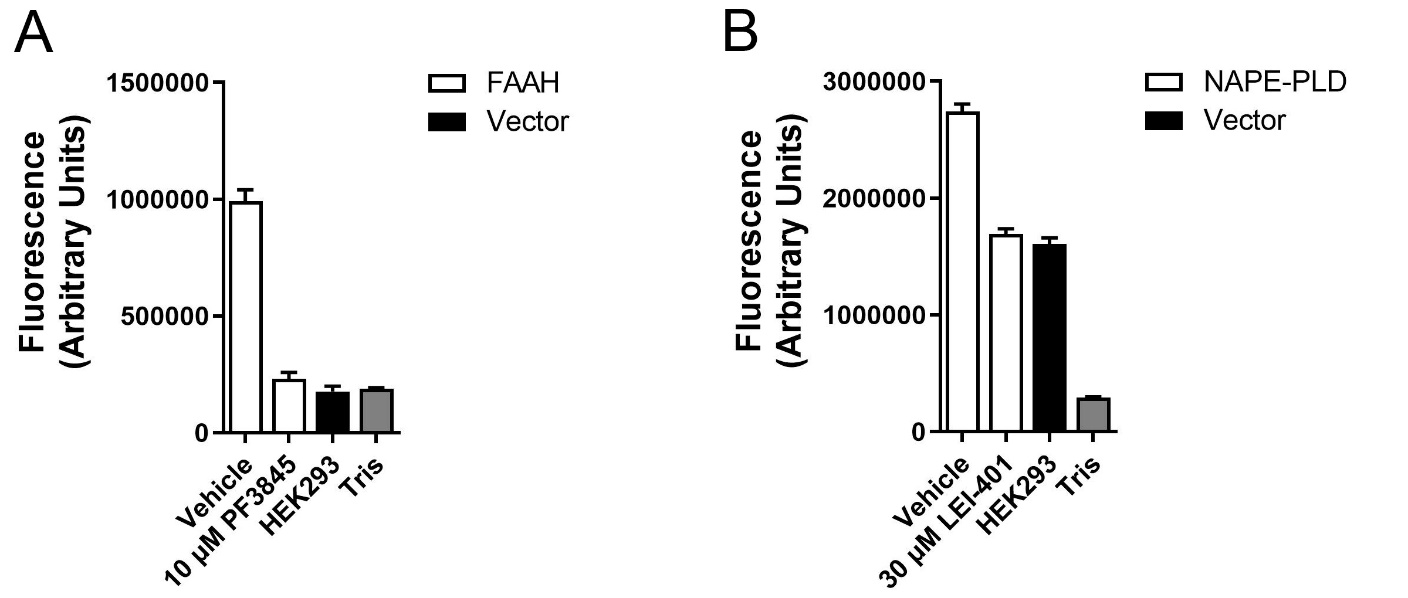


**Supplementary Figure 3.** Validation of FAAH and NAPE-PLD activity assays. (**a**) Hydrolysis of AMC-AEA (100 µM) by HEK293 cells transfected with mouse FAAH (white bars) or vector control (black bar). Negative control reactions only contained AMC-AEA in Tris buffer (gray bar). Note that AMC-AEA hydrolysis is only observed in FAAH transfected cells and is inhibited by PF3845 (10 µM), confirming that AMC-AEA is a selective FAAH substrate (n = 3). (**b**) PED-A1 (10 µM) hydrolysis by mouse NAPE-PLD transfected HEK293 cells (white bars) incubated with vehicle (1% DMSO) or LEI-401 (30 µM) as well as vector transfected controls (black bar). Note that hydrolysis of the phospholipase A1 substrate PED-A1 is observed in vector transfected cells. Importantly, increased PED-A1 hydrolysis is observed in NAPE-PLD expressing cells and is inhibited by the selective NAPE-PLD inhibitor LEI-401. Consequently, the enzymatic activity sensitive to LEI-401 corresponds to NAPE-PLD (n = 3).


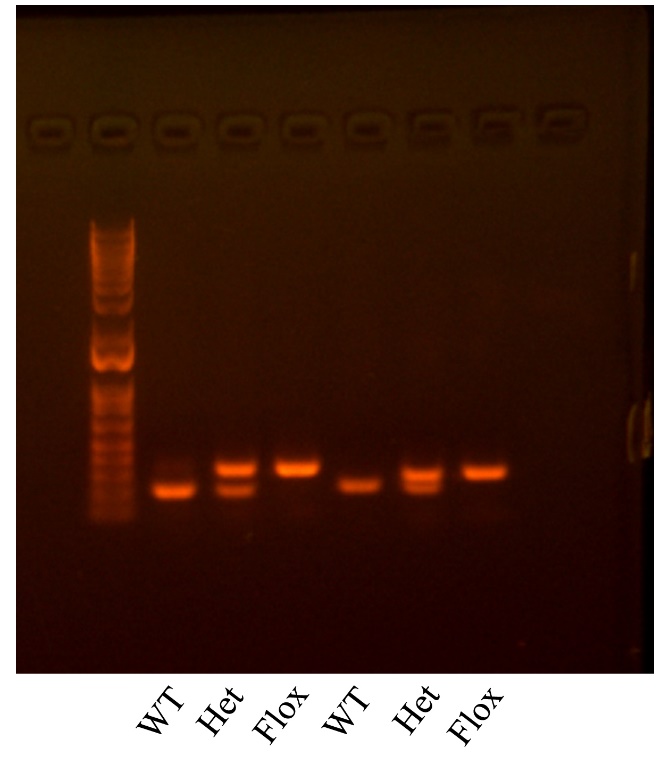


**Supplementary Figure 4.** Full image of gel presented in Fig. 1b.
